# Micro-Encapsulation of Phytochemicals in Passion Fruit Peel Waste Generated on an Organic Farm: Effect of Carriers on the Quality of Encapsulated Powders and Potential for Value-Addition

**DOI:** 10.3390/antiox11081579

**Published:** 2022-08-15

**Authors:** Gift Kabelo Kobo, Tafadzwa Kaseke, Olaniyi Amos Fawole

**Affiliations:** 1 Postharvest Research Laboratory, Department of Botany and Plant Biotechnology, Faculty of Science, University of Johannesburg, Johannesburg 2006, South Africa; 2 SARChI Postharvest Technology Research Laboratory, Africa Institute for Postharvest Technology, Faculty of AgriSciences, Stellenbosch University, Stellenbosch 7600, South Africa

**Keywords:** passion fruit peel, carriers, solubility, total phenolic content, total monomeric anthocyanin, phenolic acids, flavonoids

## Abstract

The passion (*Passiflora edulis Sims*) fruit peel is rich in phenolics and other bioactive compounds and has great potential as a natural food preservative. The present study investigated the value-adding potential of passion fruit peel waste generated on an organic farm. The effect of carriers in encapsulating the peel extract to develop a polyphenolic-rich powder was investigated. The passion fruit peel extracts were prepared using 70% ethanol (1:10 *w*/*v*), and encapsulated using waxy starch (WS), gum arabic (GA), and maltodextrin (MT) before freeze-drying. The effects of carriers on the passion fruit peel powder (PFPP) production yield, physicochemical, rheological, phytochemical, and antioxidant properties were investigated. GA-and MT-encapsulated powders had better physical, phytochemical, and antioxidant properties, including yield, total soluble solids, solubility, bulk density, total phenolic content, and ferric reducing antioxidant powder. A total of 18 metabolites, including phenolic acids (10), flavonoids (6), and stilbenes (2), were tentatively identified in all the PFPP samples, with WS exhibiting a higher concentration of the compounds compared to GA and MT. Our results indicated that no single carrier was associated with all the quality attributes; therefore, better results could be produced by compositing these carriers. Nonetheless, our results highlight the potential of passion fruit peels as a source of polyphenols and functional ingredient in formulating natural food additives.

## 1. Introduction

The fruit processing industry generates large quantities of postharvest waste, which accounts for approximately 16% of the total food waste, and 6% of the greenhouse gas emissions [1]. The global availability of these by-products and their under-exploited potential has motivated researchers to study the value-addition potential of waste from fruit processing. The seed fractions, peels, and pomace of fruit processing waste are potential raw materials for the recovery of bioactive compounds such as anthocyanins, pectin, lipids, polyphenols, and dietary fibre [1]. Commonly reported fruit-processing waste rich in bioactive compounds include grape, litchi, pineapple, citrus, apple, strawberry, papaya, pomegranate, and passion [1,2].

Passion fruit (*Passiflora edulis Sims*), belonging to the family Passifloraceae, is mainly grown in Africa, Asia, and South America, with Brazil being the largest producer and contributing between 50% and 60% of the global output [3,4]. Among the 450–500 passion fruit species, *Passiflora adulis* stands out among other species because of its economic and medicinal importance. The fruit’s medicinal properties have been linked with the treatment of different ailments in humans [5]. The current major economic importance of passion fruit lies in the production of concentrated juice, which consumes huge volumes of the fruit.

Passion fruit juice production is a viable and rapidly growing industry that generates huge quantities of by-products such as seeds and peels, which contribute to 27% and 50% of the total fruit’s weight [6]. The generated biowaste, if not reused or managed well, may become an environmental liability. The literature has shown that passion fruit peel has impressive antimicrobial, antioxidant, and antitumor properties, which are linked to the presence of bioactive compounds, including anthocyanins, ascorbic acid, flavonoids, carotenoids, and phenolics, making it a potential raw material in formulating functional food, nutraceutical, and pharmaceutical products [3,7]. Interestingly, passion fruit peel has been reported to have more functional properties than pulp, the edible portion of the fruit [8]. Nonetheless, the bioactive phytochemicals present in passion fruit peel are unstable when in contact with industrial processes and environmental conditions such as pH, high storage temperature, oxygen, light, solvents, and metal ions [9]. Therefore, strategies to protect the sensitive bioactive compounds from environmental impact and enhance their functionality are necessary.

Microencapsulation is a novel technology tailored to preserve bioactive compounds and facilitate easier handling, storage, and transportation of the bioactive components. The process involves the entrapment of valuable, sensitive or target components or fractions inside the wall material [10]. The encapsulation of plant extracts is usually carried out using carbohydrates, hydrocolloids, lipids, cellulose, and proteins-based carriers since it is impossible to produce powdered products from plant extracts without biopolymers. The selection of these encapsulating agents is based on their cost, properties, and nature of the fruit extract. Gum arabic (GA) is a hydrocolloid-based coating material used mainly due to its high-water solubility characteristics, low viscosity, and emulsifying properties [9]. Maltodextrin (MT) is a low cost, highly branched polysaccharide with high solubility that encapsulates foods with high sugar content [11]. Meanwhile, waxy starch (WS) is known for stabilizing flavours in the encapsulated product. WS, GA, and MT are among the commonly used biopolymers to encapsulate bioactive compounds from plant materials. For instance, MT alone or in combination with GA has been successfully applied to encapsulate eggplant peel extract [12], grape skin extract [9], pineapple peel extract [13], and pomegranate peel extract [14]. Meanwhile, Adetoro et al. [10] and Nthimole et al. [15] used WS to develop pomegranate, and raspberry juice powder, respectively. The authors reported varied effects of the wall materials on the developed powders’ quality; therefore, we hypothesised that these carriers had varied effects on the quality attributes of the passion fruit peel powder. The most common drying techniques available to produce encapsulated powders are freeze-drying (lyophilization) and spray drying; nonetheless, the prospective of freeze-drying is rapidly increasing because of its capability to preserve heat-labile bioactive phytochemicals, volatile compounds, and nutrients [16]. Furthermore, freeze-drying protects the sensory (taste, odour, and flavour) and physical properties of food, such as texture, appearance, shape, and colour [17].

Therefore, this research aimed to investigate the effect of GA, MT, and WS in the encapsulation of purple passion fruit peel extract to develop a freeze-dried polyphenolic-rich powder, which can be used as a natural preservative or ingredient for natural healthy foods. Furthermore, the developed powders were characterised for physicochemical, rheological, phytochemical, and antioxidant activity.

## 2. Materials and Methods

### 2.1. Materials and Chemicals

Peels from freshly processed organic passion fruit were obtained from Ganico Organic Farm, Gauteng Province, South Africa, and transported to Postharvest and Agroprocessing Research Laboratory, University of Johannesburg. Carriers including maltodextrin, gum arabic, and waxy starch were procured from Sigma Aldrich, USA, and France. Other chemicals included 2,4,6-tri(2-pyridyl)-s-triazine (TPTZ), Folin-Ciocalteu phenol reagent, gallic acid, 2,2- diphenyl-1-picrylhydrazyl (DPPH), cyanidin-3-glucoside, and 6-hydroxy-2,5,7,8-tetramethyl-chroman-2-carboxylic acid (Trolox), catechin; punicalin α and β, ellagic acid, epicatechin, punicalagin α, punicalagin β, rutin, syringic acid, chlorogenic acid, and quercetin, were of analytical grade and purchased from Sigma-Aldrich, Johannesburg, South Africa.

### 2.2. Methods

#### 2.2.1. Passion Fruit Peel Extract Preparation

The peels were cut into uniform sizes (4 × 4 mm) and then oven-dried (EcoTherm Economy, Labotec, Cape Town, South Africa) at 50 °C to a constant mass. The dried peels were ground into a fine powder (<1 mm particle size), mixed with 70% (*v*/*v*) ethanol according to Magangana et al. [18], and then sonicated using an ultrasonic cleaner (Labotec, Sonic Clean, Johannesburg, Gauteng Province, South Africa) at 0 °C for 25 min. The passion fruit peel extracts (PFPE) were filtered using Whatman filter paper No. 1 and then centrifuged (ThermoF Scientific, Biofuge, Stratos, UK) at 8400× *g* and 4 °C for 5 min. Ethanol was recovered using a BUCHI Rotavapor R-300 (Postfach, CH–9230, Flawil, Switzerland) at 60 °C. The passion fruit peel concentrated extracts were stored in darkness at 5 ± 2 °C before further analyses.

#### 2.2.2. Encapsulation and Freeze-Drying Procedure

In triplicate, the PFPE were independently mixed with GA, WS, and MT (10% *w*/*v*) at room temperature, under continuous stirring using a magnetic stirrer. The samples were then homogenised using a Stuart SHM2 homogenizer (Staffordshire, UK) for 45 s and then frozen for 24 h at −20 °C before freeze-drying in a Buchi Lyovapor L-200 Freeze Dryer (Postfach, CH–9230, Flawil, Switzerland) at −60 °C, and 0.01 mbar for 72 h. The freeze-dried samples were ground into a fine powder (<1 mm particle size), and the powder yield reported as a percentage (%) was calculated as grams of powder per 100 g of total solids in the feed solution. The PFPP was then packaged in polythene bags and stored at −20 °C and in darkness till further use.

### 2.3. Physicochemical Attributes of PFPP

#### 2.3.1. Color Measurements and Moisture Content

The moisture content of PFPP was measured at 120 °C using the moisture analyzer (KERN DBS60-3 Balingen, Germany). The color parameters, redness/greenness (a*), yellowness/blueness (b*), and lightness (L*) of dried and ground passion fruit peel and PFPP (with carrier) were measured using a CR-10 chromometer (Konica Minolta, Osaka, Japan). Respectively, Equations (1)–(3), were used to calculate the total colour (ΔE), Chroma (C*), and the hue angle (h°) [10]. All measurements were carried out in triplicate.

(1) $$\Delta E\;=[{\left(L\;-{\;L}^{*}\right)}^{2}+{\left(a\;-{\;a}^{*}\right)}^{2}+{\left(b\;-{\;b}^{*}\right)}^{2}{]}^{1/2}$$

(2) $$C*={\left({\;a\;}^{*2}+{\;b\;}^{*2}\right)}^{1/2}$$

(3) $$h{}^{\circ}={\;\tan}^{-1}\;(\frac{b^{*}}{a^{*}})$$

For ΔE, L, a, b represent colour attributes of ground passion fruit peel, while L*, a*, b* represent colour attributes for the PFPP.

#### 2.3.2. Titratable Acidity, Total Soluble Solids, and pH

In triplicate, 2.5 g of PFPP were dissolved in 2.5 mL distilled water, and vortexed for 5 min, before sonication in an ultrasonic cleaner (Labotec, Sonic Clean, Johannesburg, Gauteng Province, South Africa). The samples were then centrifuged (Thermo Fischer Scientific, Biofuge, Stratos, UK) at 8400× *g* for 25 min, and the supernatant obtained was used to determine titratable acidity (TA), total soluble solids (TSS), and pH.

TA was measured using an auto-titrator (Orion Star T910, Thermo Fisher Scientific, Sussex, UK). Briefly, the supernatant (2 mL) was mixed with 90 mL deionised water and then titrated against sodium hydroxide (0.2 N) until the pH was 8.2. The results were reported as a percentage. TSS was measured using a PT-32 refractometer (ATAGO, Tokyo, Japan), and the results were expressed as Brix. The Insmark LS128 pH meter (Mumbai, India) was used to measure pH.

### 2.4. Technofunctional Properties of PFPP

#### 2.4.1. Hygroscopicity, Solubility, and Bulk Density Determination

For hygroscopicity measurements, 2 g of the PFPP were placed in a desiccator with sodium chloride saturated solution at 25 °C (68.9% RH) for 24 h. Hygroscopicity was reported as a gram of water absorbed per 100 g of PFPP. All measurements were performed in triplicate.

To measure solubility, PFPP (0.5 g) were mixed with 50 mL deionised water, after which the solutions were vortexed for 30 s and then centrifuged using a Thermo Fisher Scientific, Biofuge centrifuge (Stratos, UK) at 756× *g* and 4 °C for 5 min. An aliquot of 12.5 mL of the supernatant was then transferred to a pre-weighed petri dish and dried in an oven for 5 h at 105 °C. Solubility was calculated using Equation (4). All measurements were performed in triplicate.

(4) $$\mathrm{Solubility}\;(\%)=\frac{\mathrm{initial}\;\mathrm{weight}-\mathrm{final}\;\mathrm{weight}}{\mathrm{Initial}\;\mathrm{weight}}\times 100$$

Bulk density was measured by determining the volume occupied by the dry powder. A 10 mL measuring cylinder was filled with 5 g of PFPP and dropping it 10 times on a bumpy surface from a height of 15 cm. Bulk density was determined using Equation (5) [10]. All measurements were carried out in triplicate.

(5) $$\mathrm{Bulk}\;\mathrm{density}\;(g/\mathrm{mL})=\frac{\mathrm{mass}\;\mathrm{of}\;\mathrm{the}\;\mathrm{powder}}{\mathrm{volume}\;\mathrm{estimate}}$$

#### 2.4.2. Oil-and Water-Holding Capacity Determination

The method described by Adetoro et al. [10] was used to determine the oil-holding capacity (OHC) and water-holding capacity (WHC). Briefly, distilled sunflower oil or water (25 mL) was mixed with PFPP (250 mg), vortexed, and centrifuged (Thermo Fisher Scientific, Biofuge, Stratos, UK) at 1344× *g* and 25 °C for 10 min. The residues were dried, weighed, and the OHC/WHC expressed as g/g was calculated as the difference in weight between the fresh residue and dry residue divided by the dry residue. All measurements were carried out in triplicate.

### 2.5. Particle Size Distribution and Morphology

#### 2.5.1. Microstructure Analysis

A TESCAN, Vega 3 scanning electron microscope (Brno, Czech Republic) was used to examine the PFPP. A thin layer of gold was applied to the uniformly mixed powders using a sputter coater, after which the coated samples were then viewed using a scanning electron microscope at 100 and 500× magnifications. The images were processed using National Institutes of Health’s (Bethesda, MD, USA) ImageJ software.

#### 2.5.2. X-ray Diffraction Analysis

Structural analysis of the freeze-dried PFPP was undertaken using X-ray diffraction (XRD) technique and X’Pert Empyrean diffractometer (PANalytical, Mawern, UK) with Cu radiation (l = 0.154) operated at a voltage and current of 40 kV, and 40 mA, respectively. The scanners were determined at 2θ with a range from 10°–80° with a step size of 0.017° using nm and 20 s per step.

### 2.6. Bioactive Compounds, Encapsulation Efficiency, and Antioxidant Activity

#### 2.6.1. Total Phenolic Content, Encapsulation Efficiency, and Total Monomeric Anthocyanin

In triplicate, PFPP (0.5 g) was separately mixed with 50% methanol (10 mL), vortexed, sonicated, and centrifuged (Thermo Fischer Scientific, Biofuge, Stratos, UK) at 8400× *g* for 25 min, and the supernatant (PFPP extracts) obtained was used for the determination of total phenolic content, total monomeric anthocyanin, and antioxidant activity.

The Folin–Ciocalteu method was used to determine the total phenolic content [19]. A mixture of 50 µLPFPE or PFPP extract, 450 µL methanol (50%), and 500 µL Folin–Ciocalteu was vortexed for 30 s and incubated for 10 min in darkness before adding 2% sodium carbonate. The mixture was then further incubated for 40 min in the dark before the absorbances were read using a SP-UV 300 UV-vis spectrophotometer (Shanghai, China) at 520 nm, using 50% methanol as a blank. Gallic acid was used to prepare the standard curve (0–10 mg/mL; R^2^ = 0.9889) and the final results were reported as mg gallic acid equivalent per mL PPFE (without carriers) or g PFPP (mg GAE/mL or g PFPP). All measurements were carried out in triplicate.

The total phenolic content encapsulation efficiency (EE) was calculated as the ratio between the total polyphenols in the reconstituted PFPP and PFPE (without carrier), according to Equation (6).

(6) $$\mathrm{EE}\;(\%)=\frac{\mathrm{TPC}\;\mathrm{in}\;\mathrm{PFPP}}{\mathrm{TPC}\;\mathrm{in}\;\mathrm{PFPE}\;\left(\mathrm{without}\;\mathrm{carrier}\right)}\times 100$$

The total monomeric anthocyanin (TMA) was determined using the method described by Fawole and Opara [19]. Briefly, PFPP extracts were separately mixed with two buffers (pH 1; potassium chloride) and (pH 4.5; sodium acetate) and the absorbances (A) for each buffer were measured at 520 and 700 nm, respectively, using a SP-UV 300 UV-vis Spectrophotometer (Shanghai, China). Using Equations (7) and (8), the TMA was calculated, and the results were reported as cyanidin-3-glucoside equivalent (C3gE) per g dry matter (mg C3gE/g DM). All measurements were carried out in triplicate.

(7) $$A=\left(A1-A2\right)\;\mathrm{pH}\;1.0-\left(A1-A2\right)\;\mathrm{pH}\;4$$

(8) $$\mathrm{TMA}\;(\mathrm{mg}/\mathrm{mL})=\frac{A\;\times \;\mathrm{MW}\;\times \;\mathrm{DF}}{\varepsilon \times \;L}$$

where A = absorbance, A1 = absorbance at 500 nm, A2 = absorbance at 700 nm, MW = anthocyanin molecular, weight (449.2), ε = cyd-3-glucoside molar absorbance (26,900), L = cell path length (1 cm), and DF = dilution factor.

#### 2.6.2. Ferric Reducing Antioxidant Power and DPPH Radical Scavenging Activity

A coulometric method described by Fawole and Opara [19] was used to determine the ferric-reducing antioxidant power (FRAP) of the PFPP. The FRAP reagent was prepared using 50 mL of 300 mM acetate buffer, 5 mL of 10 mM 2,4,6-tripyridyl-s-triazine, (TPTZ) and 5 mL of 20 mM ferric chloride (FeCl_3_) at previously warmed to 37 °C in a water bath. To the PFPP extracts (150 µL), 2.85 mL of the FRAP reagent solution was added, and the samples were incubated for 30 min in darkness. Using a SP-UV 300 UV-vis spectrophotometer (Shanghai, China) at 593 nm, the absorbances were measured. Trolox was used as the standard curve (0–10 mM; R^2^ = 0.9990), and the results were expressed as mM Trolox equivalents per g dry matter (mM TE/g DM).

The method reported by Fawole and Opara [19] was used to measure the radical scavenging ability (RSA) of the PFPP. The PFPP extracts (15 µL) were mixed with 735 µL methanol (50% *v*/*v*) and 750 µL of 0.1 mM methanolic 2,2-diphenyl-1-picryl hydrazyl (DPPH) solution. Thereafter, the samples were incubated for 30 min in darkness, and their absorbances read at 517 nm using an SP-UV 300UV-vis spectrophotometer (Shanghai, China). Trolox standard curve was linear between 0–2.0 mM (R^2^ = 0.9990) and the results were expressed as mM Trolox equivalent per g dry matter (mM TE/g DM). All measurements were performed in triplicate.

### 2.7. Liquid Chromatography Mass Spectrometry (LC-MS/MS) Analysis

The PFPP extracts for LC-MS/MS analysis and the profiling of the phenolic compounds were performed using the methods described by Magangana et al. [18]. The phenolic compounds were profiled on a Waters Synapt G2 Quadrupole time-of-flight (QTOF) mass spectrometer (MS) connected to a Waters Acquity ultra-performance liquid chromatograph (UPLC) (Waters, Milford, MA, USA). The chromatographic separation was performed on a Waters HSS T3 (2.1 × 100 mm: 1.7 μm) column at a constant temperature of 25 °C. The flow rate was 0.3 mL/min, injection volume-2 μL, mobile phase: 0.1% formic acid (solvent A) and acetonitrile (0.1% formic acid: solvent B). Gradient elution (100% *v*/*v* solvent A), (28% *v*/*v* solvent B): 1–22 min: 40% solvent B: 50 s: 100% solvent B: 1.5 min and re-equilibration to initial conditions: 4 min. To identify the compounds, the mass spectrometry ramp collision energy MSE scanning mode was employed to acquire both molecular and fragment ionization data using two channels; low collision energy at 4 V and high collision energy ramped from 20 to 60 V. From this, the molecular ionization and fragment data were obtained to enable tentative assignments of some of the chemicals identified. The tentative identities were further compared to published results. The electron spray ionization in negative mode was achieved for a scanning range of 120–1500 m/z.

#### 2.7.1. Determination of Individual Phenolic Compounds Concentration

Calibration curves were used to quantify the phenolic compounds. The principle of structure-related target analyte/standard (functional group and or chemical structure) was applied to choose a set of reference phenolic compounds. The regression coefficient (R^2^) was higher than 0.990 and indicated good linearity.

#### 2.7.2. Data Acquisition

The Mass Lynx 4.1 software (Central Analytical Facilities (CAF), Stellenbosch, South Africa) was used for data acquisition and processing. To reveal the major and subtle differences and similarities between the powder samples, a metabolomics approach was utilised. Eighteen metabolites were annotated.

### 2.8. Statistical Analysis

One-way analysis of variance (ANOVA), Statistica software v13 (TIBC, Palo Alto, CA 94304, USA) and SAS Software (SAS Enterprise Guideline 7.1, SAS Enterprise, Carrey, NC, USA) were used to analyse the data. The Duncan’s multiple-range test at 5% significance level, was used to separate the means and results are presented as mean ±SE (standard error). The Microsoft Excel software Version: 16,0,13029,20344 (Microsoft Cooperation, Washington, DC, USA) was used for graphical illustrations. The metabolomic data were normalised by using pooled averages, and for data-scaling, the auto-scaling algorithm was used prior to multivariate statistical analyses.

## 3. Results and Discussion

### 3.1. Moisture Content, Yield and Colour Attributes

The powder yield is one of the key quality indicators linked to the efficiency of freeze-drying process. The production yield of the PFPP ranged from 13.02 (WS) to 15.40% (MT) (Table 1), indicating that the yield was carrier dependent. The variation in the yield of the PFPP yield could be explained by the carriers’ varied configurations. These results agree with the findings of Adetoro et al. [10], who reported the highest yield of freeze-dried pomegranate juice powder from MT. The lower yield from WS-encapsulated powder could be due to the crystals formed by the waxy starch-based powder [20] The PFPP production yield in the present study (13.02–15.40%) was lower when compared to the yield of eggplant peel extract powder (39.58 to 66.47%) and dragon fruit peel extract powder (72.70%) developed using MT, and these dissimilarities could be ascribed to the variation in the encapsulation techniques, and the concentration of the carriers [12,21].

MC is an essential indicator of the freeze-drying efficiency and quality of the PFPP. The PFPP flow properties, compressibility, agglomerating tendency, and stability are highly dependent on MC. The MC of PFPP, which varied between 5.39 and 6.41%, conformed to the MC requirements of powder-based food products (3–10%) [22]. This suggests that all the powder samples could be stable under appropriate storage conditions. The MC of the PFPP was higher than the MC of MT-encapsulated pomegranate peel powder (3.69–4.60%) [14]; GA-encapsulated grape skin phenolic extract powder (2.41–2.57%) [9]; GA-, and MT-developed eggplant peel extract powder (2.34–4.11%) [12]. The variation in MC could be due to the differences in the drying technique, carrier concentration, nature of the plant extracts, among others. According to Table 1, GA-and MT-encapsulated powders exhibited higher MC than WS. This phenomenon can be explained by the complex chemical structures of GA and MT containing extensive hydrophilic groups that can easily bind to water molecules from the atmosphere during powder processing. Mahdavi et al. [23] observed similar behaviour for spray dried barberry extract with MT, GA, and gelatin as carriers. Contrary to our findings, Adetoro et al. [10] reported higher MC in pomegranate juice powder developed using WS than GA and MT. Given the differences in the performance of the carriers on MC, water activity could have been a better-quality indicator than MC.

**Figure 1:**
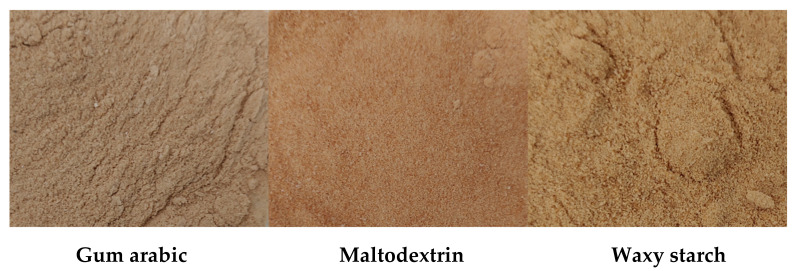
Colour of freeze-dried passion fruit peel powders developed using different carriers.

The color of PFPP is important since it can indirectly indicate the richness of the powder with bioactive pigments such as anthocyanins in the fruit peel. It was observed that the color of PFPP was carrier dependent. The PFPP color parameters; b*, a*, L*, h°, and C* are presented in Table 1. The b* (19.10–26.63) significantly (*p* < 0.05) varied among the powders, with WS exhibiting 17–28% higher values than GA and MT powders. The PFPP produced using GA and MT exhibited significantly higher parameter L* (59.87 and 61.67, respectively) than powder produced using WS (54.70), suggesting better preservation of colour pigments in the GA-and MT-encapsulated powders. Additionally, the colour of the carriers could have influenced the L* values of the powders. Higher L* was also reported in MT-encapsulated raspberry [24], orange juice [25], and mango juice powders [26]. The a* corroborate the L*, with WS-encapsulated PFPP exhibiting higher redness (22.90) than GA and MT (16.47, and 14.30, respectively). The finding that WS powder was characterised by lower h° (49.32) than GA and MT powders (53.18 and 53.43, respectively), supports the L*, a*, b* and C* results indicating that WS-encapsulated powder contained more anthocyanin compounds (Figure 1). The powders’ ΔE was calculated using passion fruit peel powder as a reference (L* = 39.63; a* = 16.13; b* = 23.87) (data not shown). The GA and MT powders showed higher ΔE than WS, which could be explained by the higher increase in the powders’ L* values compared to WS. (Table 1). This indicates that GA and MT affected the colour of the passion fruit extract more than the WS. The visually observable differences in colour of the PFPP support the ΔE results (Figure 1). Besides the carrier agents, the colour differences among the powders could be due to the oxidation of phenolic compounds in the PFPP. Factors such as particle morphology, water activity, and moisture are implicated in the browning of powder food products [27]; therefore, the larger particle sizes of the GA and MT powders could have promoted the oxidation of phenolic compounds and contributed to higher ΔE.

### 3.2. Titratable Acidity, Total Soluble Solids, and pH

**Figure 2:**
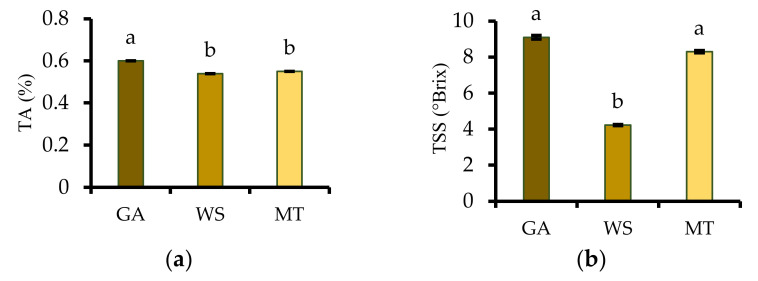

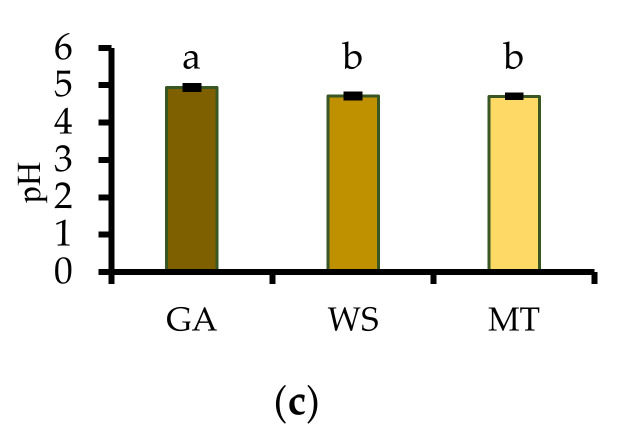
(**a**) TA (titratable acidity), (**b**) TSS (total soluble solids), and (**c**) pH of freeze-dried passion fruit peel powders. Different letters on each bar indicate significant differences in the means (*p* < 0.05). Vertical bars on each column graph indicate the standard deviation of the mean. WS = waxy starch; GA = gum arabic; and MT = maltodextrin.

TA, which is a measure of the total acids in food is an important quality determinant that can affect the food’s sensory properties. As shown in Figure 2a, GA produced PFPP exhibited significantly higher TA (0.60%) than MT (0.54%) and WS (0.55%) produced powders, which were insignificantly (*p* > 0.05) different. The results indicate that GA-encapsulated powder relatively contained more organic acids that MT and WS. The TSS of GA and MT powders were twice higher than that of WS powder. Lee et al. [28] reported that higher TSS is related to the presence of more hygroscopic compounds, which may enhance the powder’s solubility (Table 2). The pH of PFPP is valuable in controlling the availability of nutrients, biological functions, and microbial activity. According to Figure 2c, the pH of the powders narrowly ranged between 4.71 and 4.94, with GA showing significantly higher pH than WS and MT. The acidic and low pH values obtained in the present study suggest that all the powder samples could be shelf stable.

### 3.3. Solubility, Hygroscopicity, and Bulk Density

Powder solubility is a crucial quality index used to evaluate its ability to disperse in solutions. The solubility of GA-and MT-encapsulated powders was 2-3-fold higher than that of WS-encapsulated powder (Table 2), despite the GA-encapsulated powder being characterised by larger particles. Besides particle size and shape, other factors could have been implicated in the GA powder’s solubility. Overall, the smaller the particle size, the greater the surface area for hydration which increases the rate of dissolution. Similarly, Yousefi et al. [20] observed that the solubility of GA and MT-encapsulated pomegranate juice powder was 1.8 times higher than that of WS. The finding that GA- and MT-produced PFPP exhibited higher solubility implies that the powders could be easily and effectively reconstituted in water and easily incorporated and evenly distributed into other products. The higher solubility of MT-encapsulated powder was also reported with eggplant peel extract, soy sauce, sour cherry juice, mango juice, and *Moringa oleifera* leaf extract [12,29,30,31]. The low solubility of WS (30%) could affect the powder’s application, especially in reconstituted products.

Hygroscopicity, a measure of the food powder’ potential to absorb environmental moisture, is one of the primary characteristics of powder stability. According to the GEA Niro [32], powder hygroscopicity can be classified as: hygroscopic (15.1–20%), slightly hygroscopic (10–15%), and non-hygroscopic powder (<10%). Based on this classification, all the PFPP could be regarded as non-hygroscopic. This suggests that the powders would retain their flowability, and stability for an extended period, leading to longer shelf life. As shown in Table 2, the powder produced using GA was significantly (*p* < 0.05) more hygroscopic (2.13%) than the powders produced using WS and MT (2.05 and 2.07%, respectively). The findings concur with Adetoro et al. [10], who studied the hygroscopicity of freeze-dried GA, MT, and WS-encapsulated pomegranate juice. The higher hygroscopicity in GA powder could be explained by the higher water-binding capacity of GA due to the high number of ramifications with hydrophilic groups [33]. Contrary to our findings, higher hygroscopicity was reported in MT-encapsulated eggplant peel extract than GA powder [12]. The literature has reported an inverse relationship between powder hygroscopicity and MC. For instance, Tonon et al. [34] observed higher hygroscopicity in a powder that exhibited lower MC and ascribed the phenomenon to the powder’s higher moisture absorption as a result of the water concentration gradient created between the powder and the atmosphere. However, no direct relationship between these two parameters was observed in the current study. As a result, it is difficult to generalise the moisture-hygroscopicity relationship for all the powders, and each product demands an individualised approach. 

Bulk density can be used to measure the powder’s easiness of packaging, transportation, and storage. According to the literature, the bulk density of most food powders is within the range of 0.3–0.8 g/cm^3^ [28]. As shown in Table 2, the bulk density of the PFPP ranged from 0.77–0.82 g/cm^3^, indicating that the bulk density depended on the carriers. The bulk density of powder obtained in the present study was higher than eggplant peel GA-and MT-encapsulated powders (0.50–0.58 g/cm^3^) and MT-encapsulated chockberry pomace extract powder (0.65 g/cm^3^) [12,35]. While WS-produced PFPP exhibited the lowest bulk density (0.77 g/cm^3^), no significant differences were observed between the GA and MT produced powders (0.82 and 0.81 g/cm^3^, respectively). Contrasting results were reported by Caliskan and Nur Dirim [36] and Sarabandi et al. [12], who reported higher bulk density from sumac and eggplant peel extracts-encapsulated with MT than GA. Bulk density can be linked to the powder moisture content, particle size, particle-size distribution, particle shape, and inter-particle cohesion force [37,38]. Given that higher bulk density is associated with smaller particle sizes; the higher bulk density of GA and MT powders (0.81–0.82 g/cm^3^) could be due to the smaller particles observed in the respective powders. These findings suggest that the GA and MT powders might require smaller packaging, lower transport, and storage costs. Since higher MC in the powder causes the particles to stick together and increase the bulk density [39], this phenomenon can also be used to explain the higher bulk densities of GA and MT, which also exhibited higher MC (Table 1).

### 3.4. Oil-and Water-Holding Capacity

**Figure 3:**
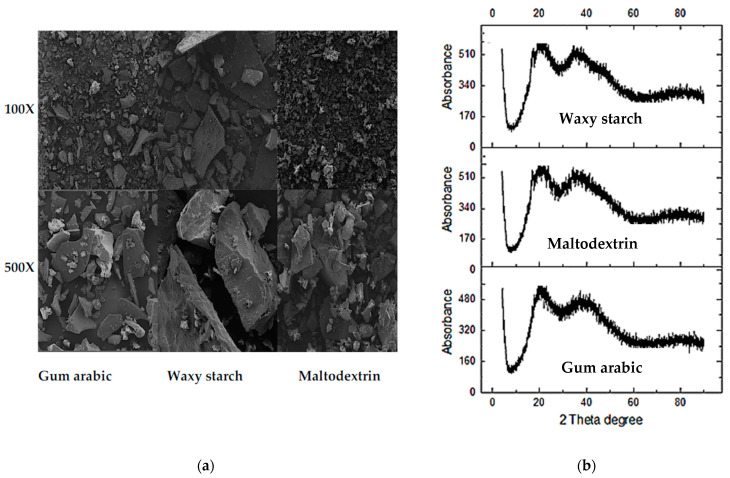
(**a**) Scanning electron microscopy micrographs, and (**b**) X-ray diffraction analysis of freeze-dried passion fruit peel powders developed using different carriers.

The OHC of the PFPP varied between 1.39 and 3.19% but insignificantly differed (*p* > 0.05) among the samples. Compared with the literature, the OHC results in the present study were higher than the OHC of GA and MT-encapsulated tomarillo juice powder (0.83–1.27%) [40]. The insignificant variation in the powders’ OHC is difficult to explain given the varied molecular weights, and chemical structures of the carrier agents, which could be expected to considerably influence the capacity of the oil to bind to the PFPP. WHC significantly (*p* < 0.05) varied among the powders (0.003–5.340%), with WS presenting the highest WHC (Table 2). In the study by Ramakrishnan et al. [40], the WHC of MT and GA-encapsulated tomarillo juice powders were 0 and 0.26%, respectively. These findings suggests that GA and MT developed powders have low WHC and could be more sensitive to storage humidity. According to the literature, WHC is related to the size and structure of the particles [41]; however, in this study, WS had the highest WHC (5.340%), whilst it was characterised by the largest and irregular particles sizes (Figure 3a). In this regard, the interpretation of WHC in this study should be made with caution.

### 3.5. Microstructures of PFPP

The particle size and morphology have an important impact on various technofunctional properties such as bulk density, compaction, flowability, solubility, and rehydration, which affect the potential applications of the powder. Scanning electron microscopy images (Figure 3a) revealed that PFPP produced using GA and WS were characterised by large (150.97–274.49 µm) (data not reported) and irregular particles. The larger and irregular particles of WS produced powder could have contributed to its lower bulk density and solubility (Table 2). Meanwhile, MT-encapsulated PFPP exhibited smaller particles (35.16–61.17 µm) (data not reported), which supports the higher bulk density and solubility observed in the respective powder (Table 2). The finding that all powder particles showed irregular shapes could be attributed to the lower drying rate and temperature of the freeze-drying process. Due to the slow drying rate, the water diffusion is slower, allowing more time for structures to deform and shrink [42]. However, both the GA and MT developed powders showed some agglomeration, where smaller particles were adhered to or formed clusters around the larger particles. Although literature has suggested that agglomeration could occur due to static electricity effects and Van der Waals forces, the presence of exposed hydrophilic groups in GA and MT could also be implicated. Overall, all powder samples showed no evidence of cracking, which could be important in the control of gas permeability and protection of the encapsulated bioactive phytochemicals.

### 3.6. X-ray Diffraction of PFPP

The x-ray diffraction (XRD) patterns of GA, MT and WS are shown in Figure 3b. The XRD patterns of the GA, MT and WS powders were similar and exhibited the strongest diffraction peaks at around 20°. This was consistent with the results of hawthorn fruit powders, which also exhibited the strongest diffraction peaks at 20° [43]. The diffraction peaks from all the powders were broad, which indicates that the crystallite size was small and that all the powders were amorphous. The literature has reported that diffuse and large peaks in amorphous materials are due to molecules that are disorderly displayed, producing dispersed bands. In contrast, crystalline materials are characterised by sharp and defined peaks because of the highly ordered molecules [44]. Other lower diffraction peaks were shown at around 38° to 40°, which further affirmed the inexistence of any ordered crystalline structure. Ersus and Yurdagel [45] studied microcapsules produced from essential oil, GA, MT, and observed that the XRD patterns of the powders demonstrated amorphous nature. Similar results were reported on MT-encapsulated and spray-dried blood fruit powder [46]. According to Liu et al. [43], smaller powder particles produce greater diffraction intensity and wider peaks; however, the current study, did not establish a clear relationship between diffraction intensity and particle size. For a clearer understanding of the relationship between particle size, diffraction intensity and crystallinity, analysing the powder particle sizes could have produced better findings.

### 3.7. Total Phenolic Content and Total Monomeric Anthocyanin

**Figure 4:**
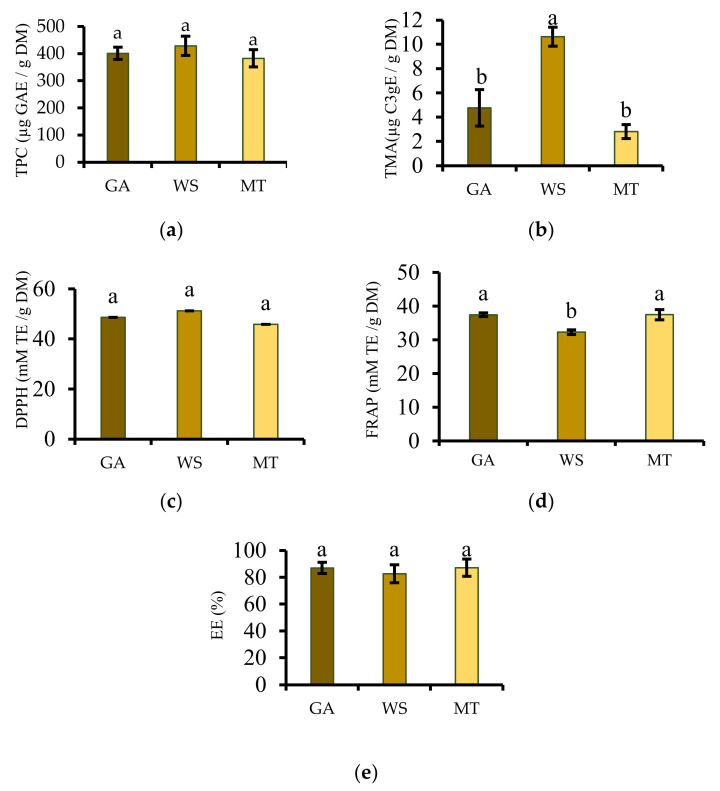
(**a**) Total phenolic content (TPC), (**b**) total monomeric anthocyanin (TMA), (**c**) DPPH radical scavenging activity, (**d**) ferric reducing antioxidant power (FRAP), and (**e**) encapsulation efficiency of the freeze-dried passion fruit peel powders. According to Duncan’s multiple-range test, different letters on each bar indicate significant differences in the means (*p* < 0.05). The standard deviation of the mean is represented by vertical bars in each column graph. MT = maltodextrin, WS = waxy starch, GA = gum arabic, and EE = encapsulation efficiency.

Phenolic compounds have antioxidant properties such as reactive oxygen species scavenging and inhibition, electrophile scavenging, and metal chelation. Therefore, TPC is one of the important attributes used to evaluate the antioxidant activity of certain plant extracts. The TPC results for the different PFPP samples (382.86–428.71 µg GAE/g DM) showed no significant differences (*p* > 0.05), suggesting that the type of carrier did not affect the entrapment of the polyphenols (Figure 4a). However, previous studies on eggplant extract, *Hibiscus sabdariffa* (Roselle) extracts, mulberry juice, *Crotalaria longirostrata* (chipilin) leaf extract, and *Arctium lappa* L. root extracts have supported GA and MT as better carrier agents to bind and preserve polyphenols from plant extracts [12,47,48,49,50].

In addition to providing plant extracts with natural colour, anthocyanins have antioxidants and anti-inflammatory properties; therefore, maximum retention of these compounds during the encapsulation process is vital for the functionality of encapsulated plant extracts. The TMA of the PFPP varied from 2.82 µg to 10.64 C3gE/g DM with WS powder showing a significantly 2–4-fold higher TMA than GA and MT powders (Figure 4b). The TMA of GA (4.78 C3gE/g DM) and MT produced powders (2.82 C3gE/g DM) were insignificantly different (*p* > 0.05). Nevertheless, different results are reported in the literature. In a previous study including GA, MT and tapioca starch, the TMA of GA-and MT-encapsulated acai powders were higher than that of tapioca starch [50]. The authors explained that the lowest concentration of TMA in tapioca starch was because of the insolubility of the material. In the study of Yousefi et al. [20], MT produced pomegranate juice powder showed higher TMA (1.24-fold higher) than GA produced powder. Furthermore, pomegranate juice powder produced using MT exhibited TMA, which was double that of WS and GA powders [10]. Notwithstanding the findings from previous studies, our results suggest that WS preserved the anthocyanin compounds better than GA and MT. It can be stated that the carriers behave differently in their ability to bind to the anthocyanin compounds depending on the type or nature of the feed product during the drying process. For a more conclusive recommendation on the effect of GA, MT, and WS on the TMA of PFPP, more operational parameters need to be investigated.

### 3.8. Antioxidant Activity

Antioxidant assays involve multiple reactions and mechanisms to determine the antioxidant activity of plant extracts; therefore, no single assay can accurately reflect the overall antioxidant activity due to the complex nature of phytochemicals. Thus, the present study employed DPPH radical scavenging activity and FRAP to evaluate if the encapsulation process affected the antioxidant activity of the bioactive compounds contained in the microparticles. As illustrated in Figure 4c, the DPPH radical-scavenging activity insignificantly varied (*p* > 0.05) among the powders, indicating that the radical scavenging activity of the powder did not depend on the carrier agent. These findings correlate with the results for TPC, which also did not significantly vary among the powders. Polyphenols have been reported to significantly contribute to the radical scavenging activity of plant extracts. Yang [48] also observed that the antioxidant activity of pomegranate peel extract powder supported the TPC results. The DPPH radical-scavenging activity insignificantly varied from 45.85 mM TE/ g DM (MT) to 51.29 mM TE/ g DM (WS) (Figure 4c), despite the literature suggesting that GA has the capacity to scavenge free radicals, chelate metal ions, and quench reactive excited states [51]. Unlike in the current study, encapsulating pomegranate juice using GA, WS, and MT, showed that MT significantly preserved the DPPH radical scavenging activity of the juice [10]. Nonetheless, in the study undertaken by Araujo et al. [52] GA-encapsulated sapota juice exhibited double the DPPH radical scavenging activity compared to MT powder. Depending on the encapsulation technique, plant extract, carrier concentration, and other factors, the performance of carriers on the radical-scavenging activity may vary.

The FRAP assay, which is based on the ability of an antioxidant to reduce Fe^3+^ to Fe^2+,^ determined that GA-and MT-encapsulated PFPP had greater antioxidant activity (37.45- and 37.47-mM TE/ g DM, respectively) than WS-encapsulated powder (32.30 mM TE/g DM), indicating that GA and MT preserved the antioxidant activity of the PFPE better than WS. However, the FRAP of GA and MT powders did not significantly differ (*p* > 0.05) (Figure 4d). These results disagree with the findings of Araujo et al. [52], who reported that GA-encapsulated sapota juice powder exhibited thrice the FRAP observed in MT developed powder. Different results were reported by Ballesteros et al. [11], who observed higher FRAP in MT produced spent coffee grounds powder (1.56 mmol Fe^2+^/100 g) than GA powder (1.21 mmol Fe^2+^/100 g). As highlighted before, the use of different assays was important in this study, given that the DPPH and the FRAP assays produced varied antioxidant activity results

### 3.9. Encapsulation Efficiency

High retention of the core materials and their minimum concentration on the surface of powder particles are desired for a successful encapsulation method. A minimum of 50% EE is desired for potential industrial scale-up [14]. According to Figure 4e, high EE ranging from 82.64 to 87.18% was obtained in the present study. The findings suggest that the polyphenols could be well accommodated in the wall material matrices. Despite the carriers having varied physicochemical properties and chemical structures that may affect the microencapsulation process efficiency, the EE in the present study insignificantly differed (*p* > 0.05) among the powders. Ribeiro et al. [53] also observed insignificant variation in EE, when they encapsulated vitamin A using GA, MT, and starch. The EE in the present study was within the range reported by Dag et al. [23] from GA-and MT-encapsulated goldenberry juice powder (77.03–84.44%). Regardless of the insignificant variation in the EE, the GA (87.00%) and MT powders (87.12%) showed slightly higher EE compared to WS powder (82.64%). Literature has supported GA and MT for higher EE; therefore, the present results could be improved by combining these two polymers.

### 3.10. PFPP Metabolomic Analysis

#### 3.10.1. Tentative Identification of Metabolites

**Figure 5:**
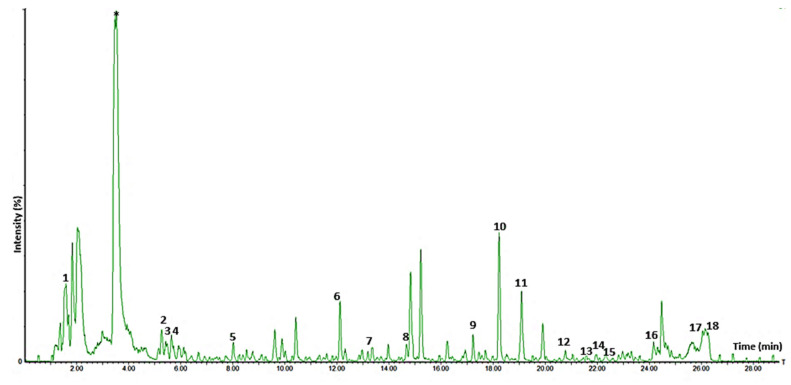
A typical liquid chromatography mass spectrometry (LC-MS) chromatograph of the individual phenolic compounds identified in the passion fruit peel powder and their retention times (RT). 1 = Caffeic acid (RT = 1.62), 2 = 1,3-di-Caffeoylquinic acid (RT = 5.29), 3 = 3,4-di-Caffeoylquinic acid (RT = 5.48), 4 = 3,5-di-Caffeoylquinic acid (RT = 5.65), 5 = Acacetin rhamnoside (RT = 8.02), 6 = Quercetin (RT = 12.13), 7 = Protocatechuic acid glucoside (RT = 13.20), 18 = 4′-Methoxyluteolin-8-C-β-D-glucopyranoside (RT = 14.66), 9 = Galloyl shikimic acid di-HHDP-glucose (RT = 17.23), 10 = Kaempferol 3,7,4′-O-triglucoside (RT = 18.22), 11 = Piceatannol I (RT = 19.09), 12 = Hydroxy gallic acid (RT = 20.77), 13 = Valoneic acid (RT = 21.87), 14 = Piceatannol II (RT = 21.98), 15 = (Epi)catechin-(epi)catechin (RT = 22.24), 16 = Vanillic acid glucoside (RT = 24.48), 17= Coumaric acid derivative (RT = 26.04), 18 = Luteolin-3-O-(6″-O-malonyl)-glucoside (RT = 26.31).* Not identified according to the methodology applied.

**Figure 6:**
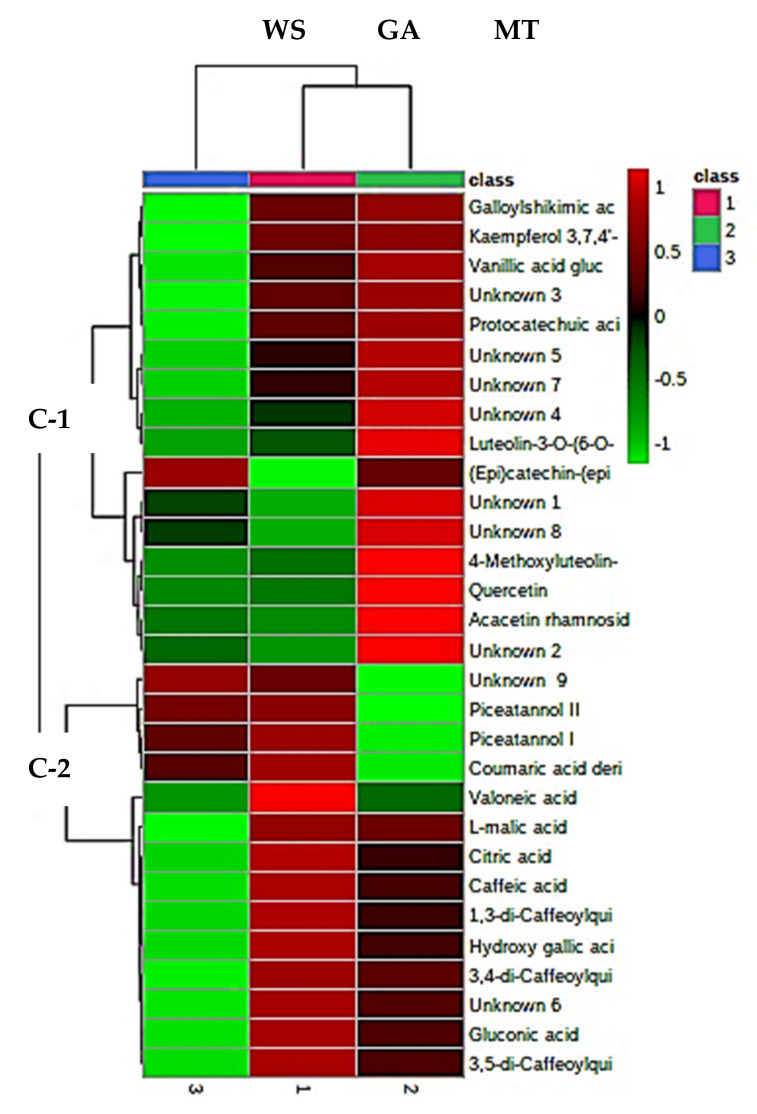
Heat map illustrating the relative concentrations of the identified compounds from the freeze-dried passion fruit peel powders developed using different carriers. The red boxes indicate relatively higher mean concentrations, while the green boxes indicate lower concentrations among the powders. GA = gum arabic, MT = maltodextrin, WS = waxy starch.

In the current study, 18 phenolic compounds, including phenolic acids (10), flavonoids (5), and stilbenes (2), were tentatively identified and characterised in all the PFPP (Supplementary Materials Table S1 and Figure 5). The relative concentrations of the identified phenolic compounds for each carrier are illustrated in a heat map, in which the variation in the metabolites’ concentrations are denoted by different colours, with red colour representing high concentrations, while low concentrations are denoted by a green colour (Figure 6). The results showed that the concentration of metabolites notably varied among the powders. The phenolic compounds identified in this study are similar to those reported in the literature [54]. The proportion (%) of each class of the metabolites was illustrated visually using a pie chart, calculated as the number of compounds per class divided by the total number of phenolic compounds identified.

**Figure 7:**
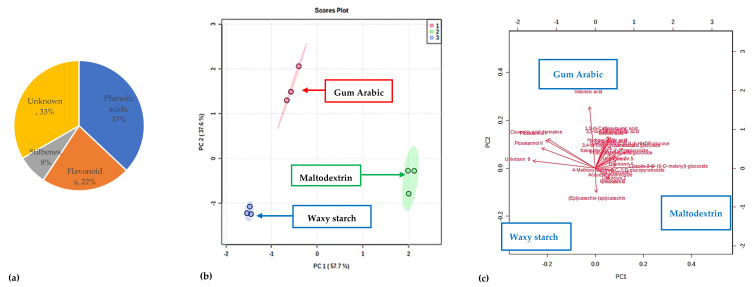
(**a**) Classification and proportion of the metabolites identified in the passion fruit peel powders, (**b**) principal component analysis (PCA) score plot and (**c**) PCA biplot showing potential biomarkers linked to the differences noted in passion fruit peel extracts encapsulated with gum arabic, maltodextrin, and waxy starch.

Phenolic acids (ca 37% of total) were detected as the majority phenolic compounds group in the PFPP (Figure 7a). Caffeic acid was the first eluted (RT = 1.62) and most abundant phenolic acid. Chlorogenic acids, including the derivatives of caffeoylquinic acid (3,4-di-Caffeoylquinic acid, 1,3-di-Caffeoylquinic acid, and 3,5-di-Caffeoylquinic acid) were also detected but in minor quantities (Figure 5). The other phenolic acid compounds included tannins (valoneic acid, and vallinic acid glucoside), phenolic acid glucosides (protocatechuic acid glucoside, and galloyl shikimic acid di-HHDP-glucose), hydroxy gallic acid (derivative of gallic acid), and a coumaric acid derivative. Flavonoids have been detected in almost every part of the passion plant, including the leaves, pulp, and peel [55], and these compounds have been reported to possess health-promoting properties [56]. In the current study, the identified flavonoids (ca 22% of total) were classified as flavanols (epi)catechin-(epi)catechin, quercetin), flavones (acacetin rhamnoside) and flavonoid glycosides (4′-Methoxyluteolin-8-C-β-D-glucopyranoside, kaempferol-3,7,4′-O-triglucoside,luteolin-3-O-(6″-O-malonyl)-glucoside). In addition, two stilbene compounds were identified, which were piceatannol I and piceatannol II, and these were eluted at 19.09 and 21.98 min, respectively. The literature has revealed that stilbenes are limited to a few foods such as grapes, red wine, and peanuts; therefore, the presence of these compounds in the PFPP is an important development for the potential application of PFPP as an ingredient for functional foods. The stilbenes have been suggested to provide protection against chronic diseases, including cancer, cardiovascular and neurodegenerative pathologies [57]. About nine compounds (ca 33% of total) with retention times varying from 9.62 to 22.91 min could not be identified during the LC-MS analysis.

To visualize the distribution of the metabolites, principal component analysis was performed. The principal component (PC) 1 could explain 57.7% of the total data variability, while PC 2 could explain 37.6% of the total variance (Figure 7b). Based on the literature, PC1 and PC2 normally explains the primary and minor variability on the x and y-axis, respectively. The score plot grouped the powders into three separate groups: namely waxy starch (blue colour), maltodextrin (green colour), and gum arabic (red colour) (Figure 7b). However, more of the metabolites were associated with GA and MT, than WS powder. Identification of the metabolites mainly responsible for the clustering (Figure 7b), was possible through the construction of a biplot (Figure 7c). About eight metabolites were identified as the discriminants of the three groupings of the PFPP: valoneic acid, coumaric acid derivative (phenolic acids), luteolin-3-O-(6″-O-malonyl)-glucoside, (Epi)catechin-(Epi) catechin) (flavonoids), piceatannol I, piceatannol II (stilbenes) and unknown 9 (with [M-H]- at *m*/*z*, and retention time of 485.1211, and 22.91, respectively (Supplementary Materials Table S1).

#### 3.10.2. Quantification of Some of the Metabolites

Quantitative analysis of the phenolic acids (caffeic acid, 1,3 di-Caffeoylquinic acid, 3,5 di-Caffeoylquinic acid, 3,4 di-Caffeoylquinic acid, coumaric acid derivative, and flavonoids (quercetin, and (Epi)catechin-(epi)catechin) was undertaken, and the results are presented in Table 3. Caffeic acid concentration ranged from 55.36 (MT) to 57.55 µg/g (WS) but did not significantly vary among the powders. Compared to other fruit peel powders, the concentration of caffeic acid for the PFPP was higher than the one reported from MT-encapsulated pineapple peel powder [13]. Meanwhile, the concentration of caffeic acid derivatives, including 1,3 di-Caffeoylquinic acid, 3,4 di-Caffeoylquinic acid, and 3,5 di-Caffeoylquinic acid significantly (*p* < 0.05) varied among the powders. For instance, 1,3 di-Caffeoylquinic acid and 3,5 di-Caffeoylquinic acid were significantly higher (29.40 and 19.23 µg/g, respectively) in GA- encapsulated powder than MT (26.96 and 18.64 µg/g, respectively) and WS (26.44 and 18.49 µg/g, respectively). However, 3,4 di-Caffeoylquinic acid was 18–27% higher in WS than GA and MT powders. Another phenolic acid, a coumaric acid derivative, which was only detected in GA and WS, was 12% higher in WS-encapsulated powder than GA. Among the phenolic compounds quantified, quercetin was the most predominant and ranged between 245.76 and 433.79 µg/g, with WS exhibiting significantly higher (16–43% higher) concentrations than MT and GA (Table 3). The quercetin can therefore be considered one of the important biomarkers for the bioactivity of PFPP. The observation that quercetin was considerably higher (*p* > 0.05) in the WS-encapsulated powder than the GA- and MT-produced powder disagrees with the TPC results, which did not significantly vary among the powders; however, a better conclusion could have been reached through the quantification of a wide spectrum of the individual phenolic compounds. (Epi)catechin-(epi)catechin was 39–62% higher in WS powder than GA and MT.

## 4. Conclusions

The study evaluated the yield and quality of passion fruit peel powders produced using GA, MT, and WS as carriers. Based on our results we established that the quality of passion fruit peel powders was carrier dependent. These results are important to potential processors of passion fruit peel waste into value-added products such as powders in terms of selecting the best carrier to retain the phenolic compounds and possess better physicochemical and technofunctional properties. The powder produced with GA and MT showed better physical, phytochemical, and antioxidant properties, which included yield, TSS, solubility, bulk density, TPC, FRAP, and colour. These quality attributes are desired for the formulation of functional foods and natural food additives by the food industry. The 18 phenolic compounds that included phenolic acids, flavonoids, and stilbenes varied among the powders, with WS exhibiting a higher concentration of the compounds compared to GA and MT. Our results indicated that no single carrier could exhibit all the quality attributes; therefore, compositing these carriers would produce better results. Additionally, further studies to evaluate the potential of the PFPP as a natural food preservative are recommended. In addition, vitro digestibility, bioavailability, bioaccessibility, and toxicological studies are necessary, given the potential application of the powders as additives in functional foods.

## Figures and Tables

**Table 1:** Moisture content (MC), yield (%) and color of freeze-dried passion fruit peel powders obtained with GA, WS, and MT as carries.

Carrier	MC (%)	Yield (%)	L*	a*	b*	h°	C*	ΔE
GA	6.41 ± 0.35 ^a^	14.65 ± 1.35 ^b^	59.87 ± 0.85 ^a^	14.30 ± 0.17 ^c^	19.10 ± 0.12 ^c^	53.18 ± 0.33 ^a^	23.86 ± 0.10 ^c^	21.03 ± 1.62 ^ab^
WS	5.39 ± 0.14 ^b^	13.02 ± 1.34 ^c^	54.70 ± 0.47 ^b^	22.90 ± 0.49 ^a^	26.63 ± 0.02 ^a^	49.32 ± 0.50 ^b^	35.13 ± 0.44 ^a^	17.06 ± 0.33 ^b^
MT	6.10 ± 0.23 ^a^	15.40 ± 1.86 ^a^	61.67 ± 1.08 ^a^	16.47 ± 0.09 ^b^	22.20 ± 0.21 ^b^	53.43 ± 0.11 ^a^	27.64 ± 0.38 ^b^	22.29 ± 1.66 ^a^

Values represent mean ± SE of triplicate determinations. In each column, statistical significance (*p* < 0.05) is indicated by different superscript letters according to the Duncan multiple range test. C* = chroma; ΔE = total color difference; L* = lightness; h° = hue angle; a* = redness; b* = yellowness; GA = gum arabic; WS = waxy starch; MT = maltodextrin.

**Table 2:** Techno-functional properties of passion fruit peel freeze-dried powders obtained with MT, WS, and GA as carriers.

Carrier	Hygroscopicity (%)	WHC (%)	OHC (%)	Bulk Density (g/cm^3^)	Solubility (%)
GA	2.13 ± 0.032 ^a^	0.003 ± 0.001 ^c^	1.42 ±0.11 ^a^	0.82 ± 0.002 ^a^	91.33 ± 3.53 ^a^
WS	2.05 ± 0.001 ^b^	5.340 ± 0.374 ^a^	3.19 ± 1.24 ^a^	0.77 ± 0.002 ^b^	30.67± 5.46 ^b^
MT	2.07 ± 0.009 ^ab^	1.938 ± 0.015 ^b^	1.39 ± 0.15 ^a^	0.81 ± 0.004 ^a^	76.00 ± 3.46 ^a^

Values represent mean ± SE of triplicate determinations. According to Duncan multiple range test, different superscripts in each column indicate statistical significance (*p* < 0.05). WS = waxy starch GA = gum arabic; and MT = maltodextrin; OHC = oil-holding capacity; WHC = water-holding capacity.

**Table 3:** Concentration of selected phenolic compounds from the freeze-dried passion fruit peel powders developed using different carriers.

Phenolic Compound (µg/g)	Gum Arabic	Maltodextrin	Waxy Starch
Phenolic acids			
Caffeic acid *	56.56 ± 1.55 ^a^	55.36 ± 5.58 ^a^	57.55 ± 1.40 ^a^
1,3 di-Caffeoylquinic acid ^A^	29.40 ± 2.09 ^a^	26.96 ± 1.32 ^b^	26.44 ± 0.18 ^b^
3,4 di-Caffeoylquinic acid ^A^	20.15 ± 0.11 ^c^	22.62 ± 1.77 ^b^	27.51 ± 0.74 ^a^
3,5 di-Caffeoylquinic acid ^A^	19.23 ± 0.39 ^a^	18.64 ± 0.19 ^b^	18.49 ± 0.25 ^b^
Coumaric acid derivative ^B^	241.97 ± 12.93 ^b^	ND	272.09 ± 22.47 ^a^
Flavonoid			
Quercetin *	245.76 ± 3.03 ^c^	362.47 ± 29.34 ^b^	433.79 ± 5.52 ^a^
(Epi)catechin-(Epi)catechin ^C^	76.82 ± 5.51 ^c^	123.26 ± 3.11 ^b^	200.63 ± 2.54 ^a^

Values represent mean ±SE of triplicate determinations; Different superscripts ^(a–c)^ in each row are significantly different (*p* < 0.05); ND = not detected. ^A^ Expressed in equivalents of caffeic acid ^B^ Expressed in equivalents of *p*-coumaric acid ^C^ Expressed in equivalents of catechin * Confirmed using a pure chemical standard.

## Data Availability

Data is contained within the article or Supplementary Materials.

## Supplementary Materials

The following supporting information can be downloaded at: https://www.mdpi.com/article/10.3390/antiox11081579/s1, Table S1: Individual phenolic and flavonoid concentration in passion fruit peel powder developed using different carriers (gum arabic, maltodextrin, and waxy starch).
