# Supplementary material for: Micro-Encapsulation of Phytochemicals in Passion Fruit Peel Waste Generated on an Organic Farm: Effect of Carriers on the Quality of Encapsulated Powders and Potential for Value-Addition
Source: Antioxidants (Basel). 2022 Aug 15;11(8):1579. doi: 10.3390/antiox11081579 (PMC9404774; doi:10.3390/antiox11081579)
Supplement: Supplementary file 1 [file antioxidants-11-01579-s001.zip › antioxidants-1844340-supplementary.pdf]

Table S1. Individual phenolic and flavonoid concentration in passion fruit peel powder developed using different carriers (gum arabic, maltodextrin, and waxy starch).

No.	Tentative ID	Retention time	M-H	MS^E Fragment ions	UV max	Elemental formula
				**Phenolic acids**		
1	Caffeic acid*	1.62	179.0562	**266.0874**, 215.0311, 176.0561, 104.0345, 96.9650, 74.0235	229	$C_8H_3O_5$
2	1,3-di-Caffeoylquinic acid	5.29	515.1234	**515.1203,** 353.0007, 341.1035, 191.0888, 179.0355, 173.0070, 135.6668, 111.0076, 89.0255	229; 262	$C_{25}H_{23}O_{12}$
3	3,4-di-Caffeoylquinic acid	5.48	515.1295	**515.1351,** 473.1112, 353.3334, 341.1131, 335.0388, 191.0004, 179. 0988, 173.0093, 135.6668, 111.0077	229; 274	$C_{25}H_{23}O_{12}$
4	3,5-di-Caffeoylquinic acid	5.65	515.1205	**515.1243**, 353.0445, 300.0888, 271.8899, 191.0999, 179.0991, 173.0996, 135.0665, 111.0071	229; 274	$C_{25}H_{23}O_{12}$
5	Protocatechuic acid glucoside	13.20	315.1075	**243.1686**, 592.1250, 349.0259, 331.1505, 205.0503, 167.0382, 135.0468, 101.0243, 89.0237	229	$C_{13}H_{16}O_9$
6	Galloyl shikimic acid di-HHDP-glucose	17.23	311.1096	**311.223,** 179.0888, 149.8980	325	$C_{14}H_{15}O_9$
7	Hydroxy gallic acid	20.77	187.0963	**125.0929**, 579.4675, 332.8404, 301.0642, 257.0844, 209.0735, 190.9932, 177.0537, 113.0179, 89.0220, 59.0104	229	$C_7H_6O_5$
8	Valoneic acid	21.87	469.2275	**469.4345**, 315.0480, 300.0211, 277.1638, 243.0618, 159.0445, 89.0216	229	-
9	Coumaric acid derivative	26.04	265.1458	**265.1475**, 517.3561, 311.1669, 183.0117, 96.9590	229	$C_{15}H_{21}O_4$
10	Vanillic acid glucoside	24.48	329.2294	**329.2326**, 253.2153, 187.0959, 171.1025, 152.9954, 125.0953	229	$C_{18}H_{35}O_5$
				**Flavonoids**		
11	(Epi)catechin-(epi)catechin	22.24	575.1392	**575.1748**, 285.0396, 301.0350, 257.0447	229	$C_{30}H_{23}O_{12}$
12	Acacetin rhamnoside	8.02	429.0858	**151.0260,** 429.0883, 283.0690	229; 271	$C_{14}H_{21}O_{15}$
13	Quercetin*	12.13	301.0920	**95.0498**, 139.0387, 152.0120, 301.0875, 161.0395, 71.0147	229; 279	$C_{13}H_9O_7$
14	4′-Methoxyluteolin-8-C-β-D-glucopyranoside	14.66	461.1633	**101.0237**, 269.1015, 195.0653, 161.0461, 71.0105	229	$C_{19}H_9O_{14}$
15	Kaempferol 3,7,4′-O-triglucoside	18.22	761.2019	**230.0652**, 336.1085, 161.0439, 147.0439, 59.0129	229	$C_{33}H_{40}O_{21}$
16	Luteolin-3-O-(6″-O-malonyl)-glucoside	26.31	533.3900	**533.6756,** 327.2171, 287.0389, 179.0996, 161.0904, 111.6158, 101.0230	229	-

			**Stilbenes**			
17	Piceatannol I	19.09	243.0652	**243.0662**, 201.0544, 159.0457	229; 282	$C_6H_{15}O_{23}$
18	Piceatannol II	21.98	243.0660	**300.0239**, 285.0355, 265.0533, 161.0458, 133.0710, 113.0280	229	$C_5H_7O_{11}$
			**Unknown compounds**			
19	Unknown 1	9.62	219.0495	**111.0078**, 173.0123, 67.0175	229; 283	$C_{15}H_7O_2$
20	Unknown 2	10.42	313.0933	**101.0222**, 269.0965, 161.0469, 313.0930, 71.0126	229; 262	$C_{12}H_{17}O_8$
21	Unknown 3	12.97	456.1484	**119.0369**, 456.1532, 161.0421, 89.0225, 85.0304	229; 281	-
22	Unknown 4	13.35	355.1017	**554.2530**, 165.0511, 161.0486, 139.0370, 113.0199	229	$C_{23}H_{15}O_4$
23	Unknown 5	13.97	439.1762	**163.0601**, 205.0735, 119.0329, 89.0236, 59.0139	229; 262	$C_{13}H_{11}O_{17}$
24	Unknown 6	14.84	340.1030	**161.0436**, 281.1368, 113.0221, 101.0217	229; 261	-
25	Unknown 7	15.22	486.1611	**307.1017**, 440.1565, 163.0590, 125.0230, 103.0388, 59.0116	229	-
25	Unknown 8	19.91	455.2090	**263.1495**, 161.0415, 101.0242, 89.0229, 59.0122	229	$C_{31}H_3O_5$
27	Unknown 9	22.91	485.1211	**485.1206**, 375.0853, 109.0279	229; 326	$C_{35}H_{17}O_3$

*Confirmed using a pure chemical standard;- = unknown or no records in literature sources to our knowledge. Literature sources and *standards were used to corroborate existing observations. MS^E fragments in bold type face refer to the base peak (the highest peak).
